# Impact of hematocrit on measurements of the intrinsic brain

**DOI:** 10.3389/fnins.2014.00452

**Published:** 2015-01-20

**Authors:** Zhen Yang, R. Cameron Craddock, Michael P. Milham

**Affiliations:** ^1^Center for the Developing Brain, Child Mind InstituteNew York, NY, USA; ^2^Nathan Kline Institute for Psychiatric ResearchOrangeburg, NY, USA

**Keywords:** BOLD, resting-state, fMRI, hematorcrit, hemoglobin, default network, dorsal attention network, salience network

## Abstract

Blood oxygenation level dependent (BOLD)–based functional MRI (fMRI) is a widely utilized neuroimaging technique for mapping brain function. Hematocrit (HCT), a global hematologic marker of the amount of hemoglobin in blood, is known to impact task-evoked BOLD activation. Yet, its impact on resting-state fMRI (R-fMRI) measures has not been characterized. We address this gap by testing for associations between HCT level and inter-individual variation in commonly employed R-fMRI indices of intrinsic brain function from 45 healthy adults. Given known sex differences in HCT, we also examined potential sex differences. Variation in baseline HCT among individuals were associated with regional differences in four of the six intrinsic brain indices examined. Portions of the default (anterior cingulate cortex/medial prefrontal cortex: ACC/MPFC), dorsal attention (intraparietal sulcus), and salience (insular and opercular cortex) network showed relationships with HCT for two measures. The relationships within MPFC, as well as visual and cerebellar networks, were modulated by sex. These results suggest that inter-individual variations in HCT can serve as a source of variations in R-fMRI derivatives at a regional level. Future work is needed to delineate whether this association is attributable to neural or non-neuronal source of variations and whether these effects are related to acute or chronic differences in HCT level.

## Introduction

Blood oxygenation level dependent (BOLD) contrast functional magnetic resonance imaging (fMRI) is one of the most widely utilized non-invasive imaging techniques that indirectly measure brain functions. In particular, BOLD-fMRI relies on the magnetic properties of hemoglobin (Hb)—the metalloprotein in red blood cells that transport oxygen (Ogawa et al., 1990a,b, 1992). More specifically, the BOLD signal indexes the total amount of deoxygenated hemoglobin (deoxy-Hb) present in a given brain area, which is taken to reflect the balance between oxygen consumption and oxygen supply (Huettel et al., 2009). When oxygen is extracted from Hb to replenish metabolic expenditures associated with neural activity, regional cerebral flood flow increases; this in turn decreases local deoxy-Hb concentration and increases oxygenated hemoglobin (oxy-Hb), the net result of which is an increase in the BOLD signal (Lindauer et al., 2010). The extent to which the BOLD signal changes in response to this process is dependent on the concentration of Hb in the blood, which is commonly represented by hematocrit (HCT; the percentage of red blood cells in the whole blood volume) (Levin et al., 2001; Gustard et al., 2003). HCT is known to systematically vary with physiological/pathophysicological (Gaehtgens and Marx, 1987; Choi et al., 2006; Jae et al., 2014; Jin et al., 2014), pharmacological (Defrancisco et al., 2009; Drinka, 2013), and psychological factors (e.g., stress) (Dugue et al., 1992; Patterson et al., 1995), as well as with demographic variables (e.g., sex, age, race) (Dutton, 1979; Levin et al., 2001). As such, it may be of value to consider the potential influences of HCT on BOLD fMRI measures.

In fact, the task-based fMRI literature has a number of studies investigating the impact of HCT level on magnitude of BOLD activation. For example, Levin and colleagues have reported a positive relationship between HCT and percent signal change of the BOLD signal within primary visual cortex in response to photic stimulation in males but not females (2001). Further, manipulating the HCT level in the same male participants by isotonic saline hemodilution resulted in a reduction in BOLD activation, demonstrating a causal relationship. Using a motor task, Gustard and colleagues replicated the positive linear relationship between HCT and BOLD signal within the motor cortex (2003). These findings are consistent with animal studies showing that manipulating HCT level through hemodilution can change the T2^*^-weighted signal intensity in anesthetized rats (Lin et al., 1998a,b).

Given the relationship between HCT and task-evoked BOLD activation, along with observations linking resting state phenomena (e.g., low frequency fluctuation amplitudes) with task-based activation (Mennes et al., 2011; Kannurpatti et al., 2012), it is reasonable to expect that the variance of the resting state BOLD signal might be scaled by HCT. Consequently, analyses of inter-individual variation in some BOLD-based measures of intrinsic brain activity may be confounded by differences in HCT. While the resting-state fMRI (R-fMRI) community has investigated the potential contributions of various non-neural signals to measurements of intrinsic brain function, such as motion (Friston et al., 1996; Jenkinson et al., 2002; Van Dijk et al., 2012; Satterthwaite et al., 2013; Yan et al., 2013a; Power et al., 2014), respiratory fluctuations and cardiovascular cycles (Hu et al., 1995; Biswal et al., 1996; Glover et al., 2000; Chang and Glover, 2009; Birn, 2012), potential associations with HCT levels are yet to be considered.

Here, we address this gap, by testing for relationships between HCT and inter-individual differences in an array of commonly employed R-fMRI derivatives, including: (1) Degree Centrality (DC, the number of significant connections of a given voxel; Zuo et al., 2012); (2) Regional Homogeneity (ReHo, a voxel's coherence with its 26 neighbors; Zang et al., 2004); (3) Amplitude of Low-Frequency Fluctuations (ALFF, the power of the low frequency oscillations; Zang et al., 2007); (4) fractional ALFF (fALFF, relative predominance of low frequency fluctuations in the range <0.1 Hz; Zou et al., 2008); (5) Voxel-Mirrored Homotopic Connectivity (VMHC, inter-hemispheric connectivity; Zuo et al., 2010); and (6) Dual Regression (DR, large-scale network connectivity; Filippini et al., 2009). Among these measures, the calculation of DC, ReHo, fALFF, VMHC, and DR involve normalizing the input BOLD signals to unit variance; the calculation of ALFF does not include this normalization. Based on biophysical models of the BOLD signal, we expect HCT to modulate the signal's variance. Thus, we hypothesize that measures that include this normalization in their calculation will not be impacted by HCT, while measures that do not include this step will be impacted. The utilization of multiple univariate analytic approaches allowed us to capture various features of intrinsic activity and identify brain areas that may be impacted by HCT. Given well-established sex differences in HCT (Levin et al., 2001), we also examined whether these relationships are modulated by sex.

## Materials and methods

### Participants

The initial data included 531 participants from the Enhanced Nathan Kline Institute (NKI)/Rockland lifespan sample. Written informed consent was obtained from all participants prior to participation, as approved by the Nathan Kline Institute Institutional Review Board. Since HCT level changes dramatically across the lifespan and the normal range are different for children, adults, and aged people, we only included adults between 18 and 50 years of age to limit variability. A total of 226 participants are within this age range (42.6%), of which 180 completed the 2-day protocol and have both HCT and imaging data. The blood sample was obtained on day 1 and the R-fMRI scans on day 2 (~1–2 weeks apart; Nooner et al., 2012).

As HCT level is also affected by medication, disease status, and psychobiological factors, we carefully selected our sample to control for these factors by excluding participants who: (1) have a positive drug test (e.g., cocaine, cannabinoid); (2) have medical conditions (e.g., diabetes, hypertension); (3) are currently taking medication (see Supplementary Table 1 for a description of medications participants currently taking and the duration); (4) are currently diagnosed or had a history of psychiatric disease (e.g., substance abuse, major depression); (5) females who had their most recent menstrual period the day before blood draw; and (6) females who had menopause before the age of 40. After this stringent screening, we had a sample of 46 participants. Further outlier analyses were performed on HCT values and head motion. Participants' HCT values are all within 1.5 times the inter-quartile-range (IQR) relative to the group of the same sex and no outliers are present. No participant has over 50% of the volumes with frame-wise displacement (FD) (Power et al., 2012) exceeding 0.2 mm. One participant was excluded as a motion outlier due to mean FD above three times the IQR of the whole sample, leaving a total of 45 participants for final analysis (Mean age: 29.0 ± 10.8; males/females: 21/24; Caucasian/non-Caucasian^1^ : 25/20; mean *FD* = 0.09 ± 0.03). The number of participants excluded based on each exclusion criterion was shown in Table 1. See Supplementary Materials for a comprehensive list of drug tests, medical conditions, and psychiatric diseases that lead to participants exclusion.

**Table 1:** **The number of participants excluded according to each exclusion criterion**.

**Exclusion criteria**	**# of participants excluded**
Positive drug test	19
Medical condition	52
Currently taking medication	12
Currently diagnosed or had a history of psychiatric disease	93
Females who had the most recent menstrual period the day before blood draw	9
Females who had menopause before the age of 40	4
HCT value	0
Over 50% of the volumes with frame-wise displacement (FD) > 0.2 mm	0
Mean FD outside of 3 inter-quartile range	1

*The same participant may satisfy multiple exclusion criteria*.

### Blood sample collection

A 5 ml venous blood sample was collected at study entry during the first visit and tested between 2 and 6 h for Hb concentration and HCT level.

### MRI data acquisition

Imaging data were acquired using a 3.0 Tesla Siemens TrioTim scanner at NKI. For each participant, a 10-min resting-state functional MRI scan was acquired using multiband echo-planar imaging (EPI) sequence (900 volumes; *TR* = 645 ms; flip angle = 60°; 40 slices; voxel-size = 3.0 × 3.0 × 3.0 mm; effective *TE* = 30 ms; FOV = 222 mm). Participants were instructed to keep their eyes open and not to think about anything in particular. A high-resolution T1-weighted anatomical image was also acquired using a magnetization prepared gradient echo sequence (MPRAGE, *TR* = 1900 ms; *TE* = 2.52 ms; *TI* = 900 ms; flip angle = 9°; 176 slices; FOV = 250 mm; acquisition voxel size = 1.0 × 1.0 × 1.0 mm).

### Imaging preprocessing

Imaging data were preprocessed using an alpha version of the Configurable Pipeline for the Analysis of Connectomes (CPAC version 0.3.4, http://fcp-indi.github.io/docs/user/index.html). For each participant, image preprocessing included: (1) realignment to the mean EPI image in two-steps to correct for motion; (2) grand mean-based intensity normalization to put all time series on a common scale by normalizing the mean of all voxels (over space and time) to 10,000; (3) nuisance regression to remove variations due to head motion and physiological processes (e.g., respiration and cardiac processes). The model included linear and quadratic trends, mean signals from white matter, mean signals from cerebrospinal fluid, and the Friston-24 motion parameters (6 head motion, their values from one time point before, and the 12 corresponding squared items) (Friston et al., 1996); (4) spatial normalization to MNI space; (5) temporal band-pass filtering (0.01–0.1Hz, except for fALFF); and (6) spatial smoothing using a Gaussian kernel (FWHM = 6 mm).

Depending on the approach, spatial normalization and spatial smoothing happened either before or after the derivative was calculated (see next section for details). Spatial normalization included: (1) structural-to-standard registration using Advanced Normalization Tools (ANTs, http://www.picsl.upenn.edu/ANTS) (Avants et al., 2011), which has been demonstrated to have superior performance compared to other commonly used registration algorithms (Klein et al., 2009); (2) functional-to-structural registration using FLIRT with a 6-degrees of freedom linear transformation. This co-registration was further refined using Boundary-based Registration (BBR) implemented in FSL (Greve and Fischl, 2009); and (3) functional-to-standard registration using ANTs to warp to 2 mm standard space.

### Individual-level analysis

Based on the R-fMRI data, we computed the following six voxel-wise derivatives for each participant at the individual-level (DC was calculated in standard space and then smoothed. VMHC was calculated on smoothed data in standard space. ReHo, ALFF, fALFF, and DR was calculated in native space and then registered to MNI space and smoothed):

1. DC, a graph theory-based measure, identifies the most connected nodes (i.e., “cortical hubs”) within the whole-brain functional network (i.e., the functional connectome) (Zuo et al., 2012). To calculate DC, we first registered the functional data to MNI space and created a study-specific group mask to include voxels (in MNI space) present in at least 90% of participants. For each participant, a voxel-based graph was then generated within this mask: each voxel (2 mm) constitutes a node in the graph, and each functional connection (i.e., Pearson correlation) between a pair of voxels is an edge. By thresholding each correlation at *r* > 0.25 (equivalent to *p* < 0.0001 in the current study), this graph was represented by a binary undirected adjacency matrix. DC was calculated as the number of significant correlations between a given voxel and all other voxels.
2. ReHo, measures local coherence of intrinsic brain activities and is defined as the Kendall's coefficient of concordance (KCC) of the time series of a given voxel with those of its 26 nearest neighboring voxels (Zang et al., 2004).
3. ALFF: the standard deviation of the bandpass filtered (0.01–0.1 Hz in the present study) fMRI signal, measures the intensity of low frequency oscillations (Zang et al., 2007).
4. fALFF, the square root of the ratio of ALFF to the sum of amplitudes of the entire frequency range, measures the relative contribution of low frequency oscillations to the power of the whole detectable frequency range (Zou et al., 2008).
5. VMHC, measures functional connectivity between each pair of symmetric inter-hemispheric voxels (Zuo et al., 2010). For calculating VMHC, the functional data were first registered to a symmetric template (obtained by averaging the MNI152 template with its left-right flipped version) and smoothed to improve the correspondence between homotopic voxels. Pearson's correlation coefficient between the time series of a given voxel and that of its symmetrical inter-hemispheric counterpart was then computed and transformed to Fisher's z scores.
6. DR, measures the functional connectivity of large-scale networks (Filippini et al., 2009). We used the 10 commonly used intrinsic connectivity networks (ICNs) identified by Smith et al. (2009) as template maps to investigate individual differences in spatial configurations of these large-scale networks. DR includes two steps: firstly, *a spatial regression* using the template maps as spatial regressors in a general linear model (GLM) to find a time series associated with each map; and Secondly, *a temporal regression* using step 1 generated time series as temporal regressors to find the voxels associated with them. The templates were demeaned and the time series were unit variance normalized before running DR. For each participant, a DR map for a given template map represents subject-specific connectivity patterns of that ICN. The temporal regression that is performed as the second step of dual regression is not spatially constrained by the network templates. Thus, we may see connectivity with regions that are outside of the template. We interpret these results as regions whose connectivity with the network of interest varies as a function of HCT. Likewise, we interpret variations in regions that are within the network, as being variably connected to the network as a function of HCT. These are very similar to interpretations of seed-based correlation analysis results, but here our seed is the network.

### Group-level analysis

Across participants, we used the GLM implemented in a toolbox for Data Processing and Analysis of Brain Imaging (DPABI; http://www.rfmri.org; Yan and Zang, 2010) to test for the associations between HCT and the R-fMRI derivatives (main effect of HCT), as well as how this relationship is modulated by sex (HCT × Sex interaction). Specifically, we used the following regression model: a given R-fMRI derivative = **HCT** + **HCT × Sex** + Sex + Age + Race + mean FD + Global mean of a given R-fMRI derivative. In this regression model, the HCT column consisted of demeaned HCT values, and the HCT × Sex column was created by first coding male as 1 and female as -1 (Sex column), then multiplying HCT column with Sex column. The main effect of HCT reflects the association between HCT and R-fMRI derivatives regardless of sex (males and females are combined). A positive interaction would indicate that the correlations between HCT and R-fMRI derivatives are more positive for males than for females and a negative correlation would indicate a reversed pattern.

Sex, age, and race were included as nuisance variables because these are known factors that have an impact on HCT. Mean FD was included to control for the residual effect of head motion (Yan et al., 2013a). Although some of the measures include variance normalization in their computation at the individual level, many sources of nuisance variation will exist between subjects. To exclude this nuisance variation, the global mean of a given derivative was included in the group analysis, as a nuisance regressor, to account for residual systematic variation not accounted for in the other regressors (Yan et al., 2013b). Compared to other normalization approaches, such as global signal regression, the mean regression approach avoids introducing artifactual relationships with the global mean.

Group analyses were constrained within the same study-specific mask as the one used to calculate DC. The results were corrected for multiple comparisons using Gaussian random field theory (voxel threshold: *Z* > 2.33, cluster-level threshold: *p* < 0.05). For DR, the results were also corrected for the number of spatial masks used (*Z* > 2.33, *p* < 0.005).

## Results

### HCT results

**Figure 1:**
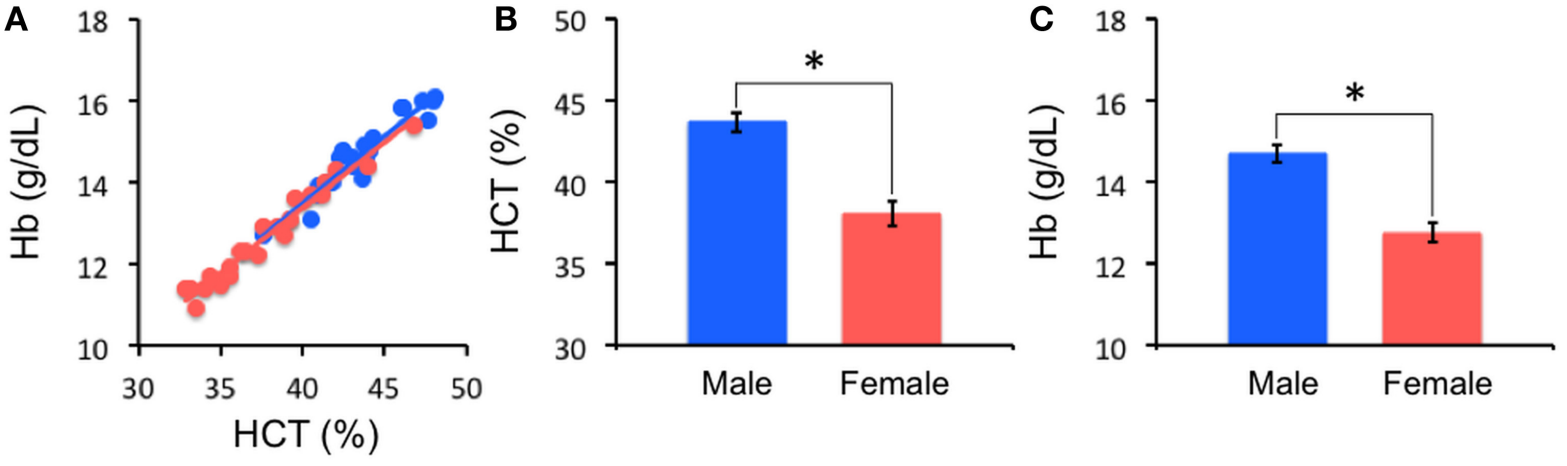
**Hematocrit (HCT) results**. HCT is highly correlated with hemoglobin (Hb) concentration **(A)**. Males (Blue) and females (Red) are significantly different in HCT **(B)** and Hb **(C)** (^*^p < 0.0001).

As expected, HCT and Hb are significantly positively correlated for both males (*r* = 0.95, *p* < 0.001) and females (*r* = 0.98, *p* < 0.001). Compared to females, males have a significantly higher level of HCT [43.68 ± 2.76 vs. 38.04 ± 3.57; *t*_(43)_ = 5.86, *p* < 0.0001, Cohen's *d* = 1.79] and Hb [14.69 ± 0.96 vs. 12.75 ± 1.16; *t*_(43)_ = 6.06, *p* < 0.0001, Cohen's *d* = 1.85] (Figure 1). In our adult sample, age is not associated with HCT (*r* = −0.08, *n* = 45; *p* = 0.60) and the effect of race on HCT levels is not significant [Caucasian: 39.87 ± 4.17; non-Caucasian: 41.67 ± 4.28; *t*_(43)_ = −1.42, *p* = 0.16, Cohen's *d* = 0.43]. No significant association between self-report first day of last menstrual period and HCT was noted in the current female sample (*r* = −0.07, *n* = 24; *p* = 0.76).

### Imaging results: global effect

We first examined whether HCT has a global effect on each of the derivatives. We found that the global means are not associated with either variables of interests (i.e., HCT and HCT × Sex), but mainly associated with age (for ReHo, DR_Executive control, DR_right_Frontoparietal) and motion (for DC, ALFF, fALFF, VMHC, DR_Visual pole, DR_Auditory. DR_right_Frontoparietal). See Supplementary Table 2 for detailed results. Thus, there is no global effect of HCT on the R-fMRI derivatives we examined.

### Imaging results: regional effect

#### The main effect of HCT

**Figure 2:**
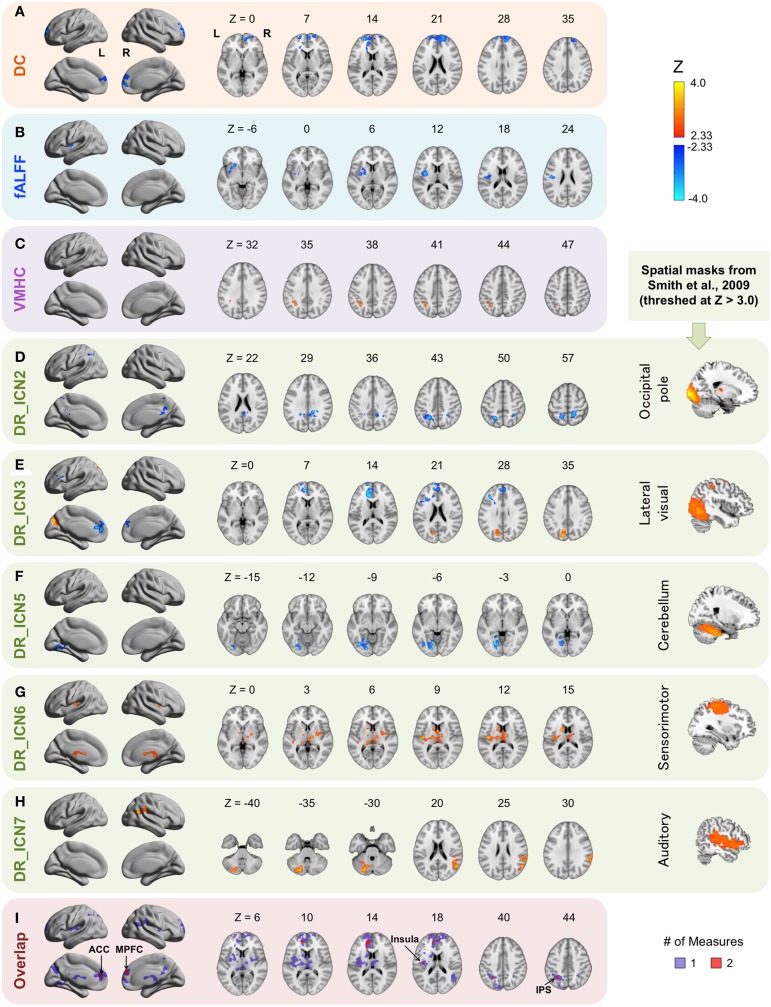
**Main effect of hematocrit (HCT)**. The Z scores for ROIs exhibiting significant main effects of HCT are plotted on surface and slice maps for Degree Centrality (DC, **A**), fractional Amplitude of Low-Frequency Fluctuations (fALFF, **B**), Voxel-Mirrored Homotopic Connectivity (VMHC, **C**), and Dual Regression (DR, **D–H**). No significant associations were detected for ALFF and Regional Homogeneity (ReHo). Surface maps were shown in lateral and medial view in MNI space using BrainNet Viewer (http://www.nitrc.org/projects/bnv/) (L, left; R, right). Locations of the axial (Z) slices were indicated in MNI coordinates. Warm colors indicate that greater values of a derivative are associated with higher HCT level (positive relationship) and cold colors indicate that greater values of a derivative score are associated with lower HCT level (negative relationship). The intrinsic connectivity networks (ICNs) used as spatial masks for DR are from Smith et al. (2009). ICN2: the occipital pole; ICN3: the lateral visual network; ICN5: the cerebellum network; ICN 6: the sensorimotor network; ICN 7: the auditory network. The slice view of these ICNs is shown on the rightmost column (for display purpose, threshed at Z > 3.0). The regional overlap across approaches is shown in **(I)**. Areas overlapping across two measures are represented by red and areas associated with one measure are represented in purple. ACC, anterior cingulate cortex; MPFC, medial prefrontal cortex; IPS, intraparietal sulcus.

The brain areas within which the intrinsic properties are associated with HCT regardless of sex were detected by the main effect of HCT (Figure 2, Table 2). Overall, four of the six R-fMRI approaches were influenced by HCT level (except for ReHo and ALFF), with the effect of DR most robust. While each R-fMRI measure revealed a distinct set of associations, overlaps were observed within anterior cingulate cortex/medial prefrontal cortex (ACC/MPFC: in DC and DR_Lateral visual), intraparietal sulcus (IPS: in VMHC and DR_Occipital pole), and insular/central opercular cortex (in fALFF and DR_Sensorimotor), suggesting that the intrinsic features of these areas may be vulnerable to the impact of HCT.

**Table 2:** **Brain areas impact by hematocrit (HCT) regardless of sex: main effect of HCT**.

**Measure**	**Region (Harvard-Oxford Anatomic Atlas)**	***BA***	**Network (Yeo et al., 2011)**	**Center of mass (MNI)**			**Volume (# of Voxels)**
				***X***	***Y***	***Z***	
DC	B Frontal pole/Paracingulate gyrus	10/32	Default	3	58	17	1856
fALFF	L Central opercular cortex/Insula/Putamen	48	Somatomotor/VA	−34	−2	9	847
VMHC	SPL/SMG	7/40	FP	−35	−50	40	207
DR_Occipital Pole	R SPL/PCC/precuneus	5/7/26	DA/Default	13	−46	41	707
	L SPL/LOC/	5/7/40	DA	−22	−55	51	710
DR_Lateral Visual	B Frontal pole/Paracingulate gyrus	10/32/48	Default	−13	43	19	1454
	L precuneus/LOC/cuneus	7/18/19	Visual	−14	−73	34	692
DR_Cerebellum	L Lingual gyrus/Fusiform gyrus	18/19	Visual	−22	−69	−6	652
DR_Sensorimotor	B Insula/Thalamus/Striatum/Brain stem	48	Somatomotor/FP/VA/Limbic	−6	−14	6	1634
DR_Auditory	L Cerebellum	−	FP/Default	−22	−73	−34	685
	R SMG/Angular gyrus/LOC	39/40/48	DA/VA/Default	54	−47	25	734

*DC, Degree Centrality; fALFF, fractional Amplitude of Low Frequency Fluctuation; VMHC, Voxel-Mirrored Homotopic Connectivity; DR, Dual Regression; B, bilateral; L, left; R, right; SPL, superior parietal lobe; SMG, supramarginal gyurs; PCC, posterior cingulate cortex; LOC, Lateral occipital cortex; BA, Brodmann Area; VA, ventral attention network; FP, frontoparietal network; DA, dorsal attention network; voxel size = 2 × 2 × 2 mm*.

Besides the areas commonly associated with two measures, several areas were associated with HCT in one measure. fALFF within the left putamen is negatively associated with HCT. The connectivity between the occipital pole network and the right posterior cingulate cortex (PCC)/precuneus and between the cerebellum network and the left lingual and fusiform gyrus are negatively associated with HCT. The connectivity between the lateral visual network and the left precuneus/cuneus, between the sensorimotor network and the bilateral insula and bilateral subcortical areas (including thalamus, striatum, and brain stem), and between the auditory network and the left cerebellum and the right temporoparietal junction are positively associated with HCT. DR measures the functional connectivity of large-scale networks, including both within- and between-network connectivity. On the whole-brain DR map, the connectivity within the original spatial template is typically strong in all subjects and the connectivity outside of this core functional architecture is more variable across subjects. We found that connections outside of the original ICN are more associated with individual differences in HCT, suggesting a larger impact of HCT on these connections.

#### The HCT × Sex interaction effect

**Figure 3:**
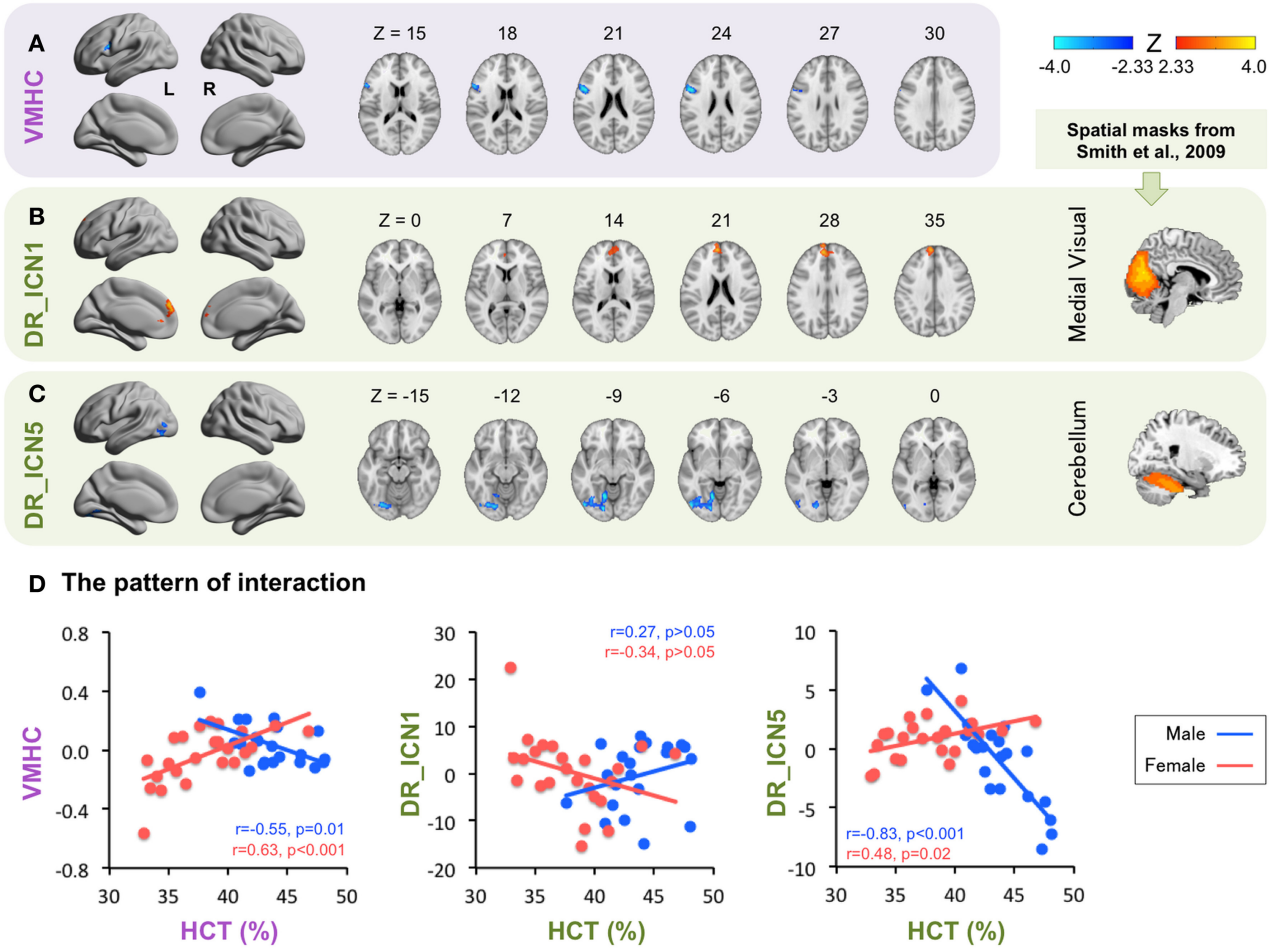
**Interaction between hematocrit (HCT) and Sex**. The *Z* scores for ROIs exhibiting significant HCT × Sex interaction are plotted on surface and slice maps for Voxel-Mirrored Homotopic Connectivity (VMHC, **A**) and Dual Regression (DR, **B,C**) in the same manner as in Figure 2. The thresholded (*Z* > 3.0) spatial maps of the intrinsic connectivity networks (ICN) used for DR are shown in the rightmost column. ICN 1, the medial visual network; ICN 5, the cerebellum network. The ROI mean derivative values are plotted as a function of HCT for males (Blue) and females (Red) separately to show the pattern of interaction **(D)**.

Consistent with the known sex differences in HCT, certain HCT effect appears to be sex-dependent (Figure 3, Table 3). Specifically, the interhemispheric connectivity (i.e., VMHC) within the pars opercularis extending into the precentral gyrus were significantly positively correlated with HCT in females (*r* = 0.63, *p* < 0.001) and negatively correlated with HCT for males (*r* = −0.55, *p* = 0.01). The connectivity between the medial visual network and the bilateral ACC/MPFC was negatively correlated with HCT for females and positively correlated with HCT for males, though both correlations did not reach statistical significance (*p* > 0.05). The connectivity between the cerebellum network and the left lateral occipital cortex, lingual gyrus, and fusiform gyrus were positively correlated with HCT for females (*r* = 0.48, *p* = 0.02) and strongly negatively correlated with HCT for males (*r* = −0.83, *p* < 0.001).

**Table 3:** **The effects of hematocrit (HCT) that are modulated by sex: HCT by sex interaction**.

**Measure**	**Region (Harvard-Oxford Anatomic Atlas)**	***BA***	**Network (Yeo et al., 2011)**	**Center of mass (MNI)**			**Volume (# of Voxels)**
				***X***	***Y***	***Z***	
VMHC	IFG (pars opercularis)/Precental gryus	44	Default/FP/DA	−52	11	22	232
DR_Medial visual	B Frontal pole/Paracingulate gyrus	9/10/32	Default	−5	54	25	658
DR_Cerebellum	L LOC/Lingual gyrus/Fusiform gyrus	18/19	Visual	−30	−74	−6	809

*VMHC, Voxel-Mirrored Homotopic Connectivity; DR, Dual Regression; B, bilateral; L, left; IFG, inferior frontal gyrus; LOC, lateral occipital cortex; BA, Brodmann Area; FP, frontoparietal network; DA, dorsal attention network; voxel size = 2 × 2 × 2 mm*.

## Discussion

We explored the possibility that variations in baseline HCT levels among individuals may have systematic associations with R-fMRI findings; this concern arises from task-based fMRI findings relating HCT and the BOLD percent signal change. Of the R-fMRI measures examined in the present work, ALFF is most similar to percent signal change (Kannurpatti et al., 2012, 2014), and was thus most likely to be impacted by HCT. Contrary to expectations, we did not find global or regional associations between HCT and ALFF, perhaps suggesting a smaller effect size for HCT-BOLD interactions than previously predicted using biophysical models (Levin et al., 2001; Gustard et al., 2003). Variance normalized measures did not show any large-scale associations with HCT either, confirming our expectations about their robustness. Interestingly, a number of regional associations were observed with the variance normalized R-fMRI indices (i.e., DC, fALFF, VMHC, and connectivity of five ICNs extracted using DR). In particular, portions of the default network (DN: ACC/MPFC), dorsal attention network (DAN: IPS), and salience network (insular and opercular cortex) showed relationships with HCT for two measures—suggesting complex interdependencies between HCT and the underlying functional architecture of these regions. Furthermore, some of the identified associations were modulated by sex.

A key question that cannot be addressed by the current observational, cross-sectional design is whether the associations observed reflect current HCT level at the time of sampling, or neural adaptations to chronic differences in HCT between individuals. This latter possibility is not without precedent. Studies of the effects of chronic hypoxia in high altitude residents have demonstrated brain functional and structural differences, which directly related to oxygen transport (Yan et al., 2010; Zhang et al., 2010). Regions affected included the prefrontal cortex, the insula, and the cingulate cortex, which are thought to be involved in cardiovascular control (Green and Paterson, 2008; Wager et al., 2009); insula and ACC are shown to be involved in the experience of dyspnea (Davenport and Vovk, 2009) and aerobic capacity (Peters et al., 2009). Also of note, rodent studies have highlighted changes in frontopolar cerebral cortex in response to chronic hypoxia in rats (Lamanna et al., 1992). In the current study, the frontal pole, ACC, insular, and IPS were each notable for exhibiting significant associations between HCT levels and two of the indices examined.

Given the close relationship between HCT level and oxygen metabolism (Ogawa et al., 1990a,b, 1992), as well as recent findings of studies focusing on the effects of chronic hypoxia on the brain (Yan et al., 2010; Zhang et al., 2010), we speculate that the involvement of these regions may be due to their roles in cardiovascular control. Alternatively, we note that the regions associated with HCT appear to follow vascular distribution of the major veins (e.g., superior sagittal sinus, transverse sinus, and sigmoid sinus) along the middle line and travel to cerebellum (Kilic and Akakin, 2008) (e.g., frontal pole, primary cortices and cerebellum); as such, it may suggest a non-neural origin. Whether these associations are due to neuronal activities or non-neuronal noises requires further delineation. Similar to the task-based literature, follow-up studies examining the impact of hemodilution-induced changes in HCT on R-fMRI indices are required to determine the extent to which the findings of the present study reflect current HCT levels as opposed to adaptation to chronic differences.

The observed associations between HCT and intrinsic brain indices may have implications for the study of inter-individual and population differences in a number of contexts. For example, a variety of experimental manipulations have been shown to impact HCT (e.g., induction of psychological stress, acute or long-term exercise training, and pharmacologic challenges) (Patterson et al., 1995; Schmidt et al., 2000; Defrancisco et al., 2009), making consideration of this variable important for efforts to increase the specificity of findings. Additionally, R-fMRI studies of participant variables, such as sex and age, would likely benefit from consideration of differences in HCT when possible as well; our findings of interactions between sex and the magnitude of associations with HCT in areas within medial prefrontal cortex, as well as visual and cerebellar networks further highlight this point. Furthermore, previous seed-based functional connectivity studies have shown that autistic (Di Martino et al., 2009) or personality (Adelstein et al., 2011) traits were more related to functional connections that were variably present across participants. Our DR results are consistent with these studies and underscore the need for future studies relating inter-individual differences in behavior to functional connectivity to take into account this hematological parameter. As R-fMRI gains popularity for studying medical populations who may have limited ambulation (e.g., stroke victims, vegetative pateints), prior findings showing the impact of posture (i.e., supine and upright) and venous stasis (Rasmussen et al., 1999) on HCT should increase motivation to account for this variable as well. Finally, investigations into variations in R-fMRI related to time of day or season may also benefit from including HCT, as it is associated with both (Thirup, 2003). Of course, in any of these scenarios, the specificity afforded by having a measurement of HCT will have to be balanced with practical considerations that impact the feasibility of obtaining blood measures.

A few noteworthy limitations exist for the present study. First, although we started with a relatively large sample, our decision to limit variability by restricting our analyses to healthy individuals resulted in a moderate sample size (*n* = 45). Besides medical and pharmacological conditions, we also excluded participants with histories of psychiatric illness to avoid potentially confounding alterations in brain-behavior relationships related to psychopathology. The moderate sample size, combined with the need for multiple comparison correction, may have limited our power to detect global effects and HCT regional effects in ALFF and ReHo. Of note, although not available yet, the Human Connectome Project dataset did include hematocrit measurements, creating the potential for future large-scale examinations of HCT. Second, as already noted, the present study was limited by its cross-sectional nature; future studies with longitudinal designs and ideally experimental manipulations (e.g., hemodilution) will be able to provide more definitive insights. Additionally, the HCT levels and imaging assessments in the present work were obtained on different days (typically within 1–2 weeks); this can add a source of unintended noise. Future work would benefit from tight coupling of HCT level assessments and MRI scanning.

Although the biophysical model predicted that HCT has an impact on BOLD signal, we did not observe an association between HCT and ALFF, the R-fMRI measure most similar to task-evoked BOLD percent signal change. We observed regional associations between baseline blood HCT level and four variance-normalized intrinsic brain measurements (i.e., DC, fALFF, VMHC, and DR), some of which are modulated by sex. Future studies that directly manipulate HCT, using hemodilution or otherwise, are necessary to differentiate whether these effects are acute or due to neural adaptations to chronic differences in HCT.

### Conflict of interest statement

The authors declare that the research was conducted in the absence of any commercial or financial relationships that could be construed as a potential conflict of interest.

## Footnotes

^1^Race was coded as “American Indian,” “Asian,” “Black,” “Native Hawaii,” “White,” and “Other” originally. Because the percentage of races other than “White” is relatively low, we combined them and recoded race as Caucasian (*n* = 25) and non-Caucasian (*n* = 20).
