# Supplementary material for: Impact of hematocrit on measurements of the intrinsic brain
Source: Front Neurosci. 2015 Jan 20;8:452. doi: 10.3389/fnins.2014.00452 (PMC4299407; doi:10.3389/fnins.2014.00452)
Supplement: Supplementary file 1 [file DataSheet1.DOCX]

**Supplementary Materials**

**Methods**

**Participants**

*A list of drug tests and the number of participants excluded according to each test*

19 participants were excluded according to 12 Drug tests (the number of participants who have positive drug tests are list in the parenthesis):

Benzodiazepines (1), Cocaine (3), Methamphetamine (0), Methadone (0), Oxycodone (0), Phencyclidine (1), Barbituates (0), THC/Cannabinoid 50 (15), Opiates (0), Tricyclic Antidepressants (0), Ecstacy (0), Amphetamines (1)

Note: two participants are positive in both Cocaine and THC/Cannabinoid 50.

*A list of medical conditions*

Participants were excluded due to health problems based on their answers to the following questions: “Does the participant have any illnesses or health problems at present? If yes, what is the condition?” For participants who answered yes, we went through their conditions and excluded 52 subjects who have the following conditions:

Anemia, diabetes, hypertension, high cholesterol, high blood pressure, hypoglycemia, cardiac ablation, cirrhosis, hypothyroidism, psoriasis, asthma, migraines, polycystic ovarian syndrome, Raynaud’s disease, mitral valve prolapse, sinus infection (current), heart murmur, chronic hepatitis C, low blood pressure, obesity, celiac disease, herniated discs, rosacea, and eczema.

*A list of psychiatric diseases*

93 participants were excluded due to psychiatric diseases (14 were currently diagnosed and 79 had a history of psychiatric diseases). A list of psychiatric diseases is below:

Anxiety Disorder, Generalized Anxiety Disorder, Posttraumatic Stress Disorder, Depressive Disorder, Major Depressive Disorder, Bipolar II Disorder; Panic Disorder Without Agoraphobia, Cannabis Abuse/Dependence, Alcohol Abuse/Dependence, Amphetamine Abuse, Cocaine Abuse/Dependence, Sedative, Hypnotic, or Anxiolytic Abuse, Opioid Dependence, Hallucinogen Abuse, Polysubstance Dependence, Social Phobia, Specific Phobia, Attention-Deficit/Hyperactivity Disorder, Obsessive-Compulsive Disorder, Eating Disorder

**Supplementary Table 1:** a description of medications currently taking and the duration.

| **Participants** | **Medications currently taking and the duration** |
| --- | --- |
| 1 | SINGULAIR: 2-5 years; Asthma pump: 2-5 years, ADVAIR: 2-5 years |
| 2 | EFFEXOR: 5-10 years; Birth control pills: 5-10 years |
| 3 | SYNTHROID: > 10 years |
| 4 | FLOVENT: 5-10 years; SINGULAIR: 5-10 years; |
| 5 | ZYRTEC: 2-5 years; VERAMYST: 2-5 years; ADVAIR: 2-5 years |
| 6 | ASMANEX: 2-5 years; SINGULAIR: 2-5 years; NASONEX: 1-2 years |
| 7 | SYNTHROID: 5-10 years; PRAVACHOL: 2-5 years; LOFIBRA: < 3 months |
| 8 | PROZAC: 5-10 years; ABILIFY: < 3 months |
| 9 | PROZAC: 6 months-1 year |
| 10 | METFORMIN: 2-5 years |
| 11 | LEVOTHYROXIN: 6 months-1 year |
| 12 | PRAVASTATIN: < 3 months; METHOCARBAMOL: < 3 months; BUPRENORPHINE: < 3 months |

Note: 12 participants were excluded due to currently taking medication based on the question: “Does the participant currently take any medications routinely?” For participants who answered yes, we checked the medications and the duration they have been taking them.

**Supplementary Table 2.** Parameter estimation on global effects of HCT.

a. 5 univariate voxel-wise approaches

| **Variable** | **Statistics** | **DC** | **ReHo** | **ALFF** | **fALFF** | **VMHC** |
| --- | --- | --- | --- | --- | --- | --- |
| **HCT** | **Beta** | -0.01 | 0.12 | 0.23 | 0.27 | 0.13 |
|  | **t** | -0.04 | 0.56 | 1.68 | 1.53 | 0.66 |
|  | **p** | -0.97 | 0.58 | 0.10 | 0.14 | 0.52 |
| **HCT × Sex** | **Beta** | 0.05 | 0.11 | 0.02 | -0.11 | -0.09 |
|  | **t** | 0.28 | 0.62 | 0.18 | -0.77 | -0.55 |
|  | **p** | 0.78 | 0.54 | 0.86 | 0.45 | 0.59 |
| **Age** | **Beta** | -0.03 | -0.35 | -0.15 | -0.26 | -0.16 |
|  | **t** | -0.20 | -2.22 | -1.40 | -1.93 | -1.01 |
|  | **p** | 0.84 | 0.03* | 0.17 | 0.06 | 0.32 |
| **Motion** | **Beta** | 0.42 | 0.01 | 0.71 | 0.59 | 0.42 |
|  | **t** | 2.79 | 0.06 | 6.88 | 4.46 | 2.73 |
|  | **p** | 0.008** | 0.95 | <0.001*** | <0.001*** | 0.009** |

b. Dual regression: intrinsic connectivity network 1 to 5.

| **Variable** | **Statistics** | **DR_Medial Visual** | **DR_Visual Pole** | **DR_Lateral_Visual** | **DR_Default** | **DR_Cerebe-llum** |
| --- | --- | --- | --- | --- | --- | --- |
| **HCT** | **Beta** | 0.31 | 0.10 | 0.02 | 0.31 | 0.11 |
|  | **t** | 1.56 | 0.49 | 0.08 | 1.44 | 0.54 |
|  | **p** | 0.13 | 0.63 | 0.94 | 0.16 | 0.59 |
| **HCT × Sex** | **Beta** | 0.06 | -0.19 | -0.06 | 0.24 | 0.04 |
|  | **t** | 0.36 | -1.16 | -0.35 | 1.42 | 0.26 |
|  | **p** | 0.72 | 0.26 | 0.73 | 0.17 | 0.80 |
| **Age** | **Beta** | -0.17 | -0.14 | -0.31 | -0.17 | -0.03 |
|  | **t** | -1.17 | -0.97 | -2.06 | -1.03 | -0.18 |
|  | **p** | 0.25 | 0.34 | 0.05 | 0.31 | 0.86 |
| **Motion** | **Beta** | 0.38 | 0.44 | 0.10 | 0.02 | 0.25 |
|  | **t** | 2.62 | 2.98 | 0.66 | 0.12 | 1.55 |
|  | **p** | 0.01 | 0.005* | 0.52 | 0.90 | 0.13 |

c. Dual regression: intrinsic connectivity network 6 to 10.

| **Variable** | **Statistics** | **DR_**  **Sensorimotor** | **DR_**  **Auditory** | **DR_Executive_Control** | **DR_Left FP** | **DR_Right FP** |
| --- | --- | --- | --- | --- | --- | --- |
| **HCT** | **Beta** | 0.26 | 0.11 | 0.17 | 0.27 | 0.25 |
|  | **t** | 1.19 | 0.57 | 0.87 | 1.27 | 1.42 |
|  | **p** | 0.24 | 0.57 | 0.39 | 0.21 | 0.16 |
| **HCT × Sex** | **Beta** | 0.08 | 0.02 | 0.08 | 0.06 | 0.001 |
|  | **t** | 0.47 | 0.11 | 0.49 | 0.37 | 0.008 |
|  | **p** | 0.64 | 0.91 | 0.63 | 0.72 | 0.99 |
| **Age** | **Beta** | -0.18 | 0.02 | -0.44 | -0.19 | -0.46 |
|  | **t** | -1.10 | 0.12 | -2.97 | -1.16 | -3.46 |
|  | **p** | 0.28 | 0.91 | 0.005* | 0.25 | 0.001** |
| **Motion** | **Beta** | -0.14 | 0.52 | 0.13 | 0.23 | 0.41 |
|  | **t** | -0.88 | 3.75 | 0.88 | 1.45 | 3.09 |
|  | **p** | 0.39 | 0.001*** | 0.38 | 0.16 | 0.004** |

Notes: The Beta, p, and t values for each variable were obtained based on the following model: Global mean of a given R-fMRI derivative = **HCT** + **HCT × Sex** + Sex + Age + Race + mean FD

HCT = hematocrit; Beta = standardized coefficients; DC = Degree Centrality; ReHo = Regional Homogeneity; ALFF = Amplitude of Low Frequency Fluctuation; fALFF = fractional ALFF; VMHC = Voxel Mirrored Homotopic Connectivity; DR = Dual Regression; FP = frontoparietal. For DC, ReHo, ALFF, fALFF, and VMHC: * < 0.05; ** < 0.01; *** < 0.001; For DR: * < 0.005; ** < 0.001; ***< 0.0001 (results were Bonferroni corrected for the number of spatial templates used).
